# Participation development of a cross-border antibiotic stewardship program as part of the EURSAFETY HEALTH-NET web 2.0 platform

**DOI:** 10.1186/1753-6561-5-S6-P155

**Published:** 2011-06-29

**Authors:** MV Limburg, R Hendrix, J Karreman, J Wentzel, LV Gemert-Pijnen

**Affiliations:** 1 Universiteit Twente, Enschede, Netherlands

## Introduction / objectives

Resistant bacteria pose more and more a risk to patient safety in Europe, especially in cross-border regions where differences in antibiotics prescription and hygiene policies exist. Antibiotic Stewardship Programs (ASPs) have been implemented in some hospitals to facilitate healthcare professionals in adequately prescribing antibiotics to improve patient safety and to reduce costs. However the implementation of ASPs does not always run smoothly. The research goal of this study is to create and implement an ASP via a participatory development process. We assume that participatory development reduces the problems associated with the implementation of ASPs.

## Methods

We have applied a literature study (non-systematic) as well as a currently ongoing ASP pilot evaluation to assess how ASPs can be implemented successfully and sustainably. Using our eHealth development roadmap, workshops with important stakeholders shall be held in the near future to further identify problems, and the stakeholders’ needs and values regarding ASP.

## Results

The literature study showed that there is a lack of usable and tailored information on antibiotic use in local care facilities. ASPs need to be implemented at a local level, based on local policies and local resistance/susceptibility patterns. These ASPs need to be carried out by multidisciplinary teams that provide prescribers with appropriate and evidence-based feedback. The implementation can include educational activities, reminders, (computer aided) decision support, and patient education, depending on the stakeholders’ needs.

## Conclusion

We expect that the ASP that is created via a participatory design process helps to realize adherence and commitment to a more prudent antibiotics prescription policy.

## Disclosure of interest

None declared.

